# Virulence determinants and antibiotic resistance in staphylococci isolated from the skin of captive bred reptiles

**DOI:** 10.1007/s11259-024-10328-w

**Published:** 2024-02-09

**Authors:** Viola Strompfová, Lucia Štempelová, Dobroslava Bujňáková, Lívia Karahutová, Mária Nagyová, Leonard Siegfried

**Affiliations:** 1 Centre of Biosciences of the Slovak Academy of Sciences, Institute of Animal Physiology, Šoltésovej 4-6, Košice, 040 01 Slovakia; 2 Faculty of Medicine, Department of Medical and Clinical Microbiology, University of P. J. Šafárik in Košice, Trieda SNP 1, Košice, 040 11 Slovakia

**Keywords:** Skin, Microbiota, Staphylococci, Reptiles, Virulence

## Abstract

Knowledge of the composition and properties of skin microbiota in healthy reptiles is essential for preservation strategies and thus the prevention of skin dysbiosis leading to dermatological diseases. Despite the greatly increasing popularity of reptiles as pets, only a few studies have dealt with this topic. Therefore, the aim of this work was to analyse species composition of bacteria isolated from skin swabs of 40 reptiles (17 species) using MALDI-TOF spectrometry and to characterise the virulence properties of identified staphylococci (*n* = 51). The most common species were *Staphylococcus xylosus* and *S. sciuri*. Bacilli, enterococci, *Escherichia coli*, *Salmonella* sp. and *Acinetobacter* sp. were also common. The most frequent antimicrobial resistance in staphylococcal isolates was observed for ampicillin (100.0%) and cefoxitin (98.0%) with the *blaZ* gene being most prevalent (58.8%). In contrast, all staphylococci were susceptible to gentamicin, kanamycin and imipenem. Slime and biofilm production was observed in 86.3% and 76.5% of isolates, respectively. Gelatinase, DNase, protease and lipase activity was found more rarely (41.2%; 25.5%; 27.5% and 21.6%). Since reptiles are a reservoir of bacteria for their owners, common multi-drug resistance (84.3%, MAR index average 0.29 ± 0.09) and biofilm formation must be kept in mind, especially in the case of injury when handling reptiles.

## Introduction

The skin constitutes the primary physical barrier between organisms and their external environment. A diverse microbial population comprising bacteria, fungi, archaea and viruses inhabit not only digestive, respiratory and reproductive tract within the human and animal bodies but also their skin (Ross et al. 2019). Although great progress in the study of the skin microbes in vertebrates by culture-independent techniques has been made in the past 15 years, reptiles have received less attention. The class Reptilia includes crocodiles, turtles, snakes and lizards. The current Reptile Database contains 12,000 species plus another 2,142 subspecies (reptile-database.org 2023). The skin structure of this animals is similar to that of mammals; on the other hand, they also have numerous differences. During evolution they developed mechanisms in order to prevent water loss and to protect against ultraviolet irradiation. Reptiles were the first organisms to evolve a *stratum corneum* with multiple layers and programmed cell death. The main special feature of their skin is that the epidermis is heavily keratinized with a layer that also prevents water loss. Scales are present but are fundamentally different from the dermal scales of fish. Two forms of keratin are produced in reptiles: α-keratin, which is flexible, and β-keratin, which provides strength and hardness and is unique to reptiles. β-keratin is found in the chelonian shells, whereas α-keratin is found in the hinges or between the scutes (Rutland et al. 2019). While in the turtles and crocodiles, the sloughing of skin is modest, in lizards, and especially in snakes, the shedding of the cornified layer, termed moulting or ecdysis, results in removal of extensive sections of the superficial epidermis. The integumental glands – one of the factors influencing microbiota colonisation – are usually restricted to certain areas of the body and play a role in reproductive behaviour or when predators are close (Kardong 2002). A further important factor is that ectothermic reptiles show considerable fluctuations and a lower average body temperature compared to most endothermic vertebrates. Despite the fact that the skin, due to its unstable temperature, pH and moisture level, is a less suitable environment for colonisation than the gut or mouth, in general, it is estimated over 10^6^ microorganisms/cm^2^ are present on human skin or other vertebrates (Ross et al. 2019). A large study using superficial skin swabs to evaluate the skin microbiome of farm, wild, zoo and household animals found that the majority of animals had higher diversity and distinct skin microbial communities when compared to human samples (Ross et al. 2018).

Since the host is an important predictor of skin microbial communities and there is lack of skin microbiome knowledge in reptiles, the present study was conducted to determine the bacterial species composition of healthy captive bred reptiles in general, focusing on staphylococci using culture-dependent techniques. Although culture-independent methods such as DNA sequencing allow an in-depth view of microbial diversity and enable the detection of microorganisms that cannot be cultivated under laboratory conditions, culture-dependent methods enable a closer look at the properties and behaviour of living bacteria. Therefore, the antibiotic resistance and virulence features of staphylococcal isolates were also tested.

### Isolation and identification of bacterial strains

Superficial swab samples (*n* = 40) from the skin of 40 healthy captive bred reptiles (17 species, Table 1) were collected from the Zoological garden in Košice (Slovakia) in April 2023. The skin swabs were collected during a routine clinical examination by a veterinarian, and the animals were not intentionally immobilised. None of the individuals showed any visual signs of illness. Amies Agar Gel Transport Swabs (Oxoid, Basingstoke, UK) soaked in sterile SCF-1 solution (50 mM Tris buffer, 1 mM EDTA and 0.5% Tween-20) were used to sample skin from the back part of the body, except the turtle due to the shell (skin on the legs was sampled). Each swab stick was rubbed on the skin 20 times (10 strokes per swab side, forward and reverse the direction of scales growth). The animal handling (fixation) and sampling was performed wearing sterile gloves for each individual. After collection, the skin swabs were stored immediately at 4 °C and processed within 3 h. The swabs were inoculated on Mannitol Salt agar, Brain Heart Infusion (both Becton and Dickinson, Sparks, USA), Pseudomonas agar (for fluorescein, HiMedia Laboratories, Mumbai, India) and MacConkey agar (Becton and Dickinson) plates within 24 h and incubated at 37 °C for 24–48 h. One or two morphologically different colonies from each sample were selected and used for species identification. The isolated bacteria were identified based on MALDI-TOF MS on a Microflex LT instrument (Bruker Daltonik GmbH, Leipzig, Germany), as described Bessede et al. (2011). To identify microorganisms, the raw spectra obtained for each isolate were imported into BioTyper software, version 2.0 (Bruker Daltonik). All staphylococcal strains were stored in the Microbank system at -̵ 70 °C till further analyses.

**Table 1:** Bacterial species identified on the skin of 40 reptiles (17 species) using MALDI-TOF spectrometry

Reptile species	Staphylococcus spp.	Other bacterial species
*Chilabothrus angulifer* (n = 4)	*S. kloosii* (2)^a^, *S. sciuri* (2), *S. xylosus* (2)	
*Eunectes notaeus* (n=1)	*S. sciuri* (1), *S. haemolyticus* (1)	
*Pogona vitticeps* (n = 2)	*S. sciuri* (2), *S. xylosus* (2)	*B. cereus* (1), *B. megaterium* (1), *E. faecalis* (1)
*Stigmochelys pardalis* (n=3)	*S. xylosus* (2), *S. arlettae* (1)	*B. megaterium* (1)
*Testudo marginata* (n = 2)	*S. xylosus* (1), *S. sciuri* (1)	*B. cereus* (2), *E. faecalis* (1), *E. casseliflavus* (1)
*Testudo horsfieldii* (n = 5)	*S. xylosus* (5), *S. cohnii* (1), *S. sciuri* (2)	*Exiguobacterium* sp. (1), *E. faecalis* (1), *E. casseliflavus* (1)
*Testudo graeca* (n = 1)	*S. sciuri* (1), *S. xylosus* (1)	
*Anolis barbatus* (n = 2)	*S. kloosii* (1)	*C. freundii* (2), *S. marcescens* (1), *B. cereus* (2), *E. cloaceae* (1)
*Uromastyx ornata* (n = 1)		*B. megaterium* (1), *B. cereus* (1)
*Eublepharis macularius* (n = 1)	*S. kloosii* (1)	*Bacillus megaterium* (1), *B. cereus* (1)
*Geochelone elegans* (n = 3)	*S. sciuri* (3), *S. kloosii* (2)	*E. coli* (1), *A. baumannii* (1), *A. radioresistens* (1),
		*B. cereus* (1), *E. faecalis* (1)
*Iguana iguana* (n = 2)	*S. xylosus* (1)	*B. kochii* (1)
*Boa constrictor* (n = 1)	*S. sciuri* (1), *S. xylosus* (1)	*P. aeruginosa* (1)
*Uromastyx acantihinura* (n = 3)		*B. endophyticus* (2), *B. oceanisediminis* (1), *B. cereus* (2),
		*B. megaterium* (1)
*Pantherophis guttatus* (n = 3)	*S. sciuri* (2), *S. xylosus* (2), *S. cohnii* (1), *S. arlettae* (1)	*B. megaterium* (1)
*Morelia spilota variegata* (n = 3)	*S. xylosus* (3), *S. cohnii* (1),	*K. marina* (1), *E. faecium* (1)
	*S. warneri* (1)	
*Elaphe schrenckii* (n = 3)	*S. xylosus* (2), *S. sciuri* (1)	*E. faecium* (3), *Salmonella sp.* (2), *B. cereus* (1), *E. coli* (1),
		*E. faecalis* (1), *B. oceanisediminis* (1)

^a^Number of animals with detected species

### Antibiotic resistance and virulence factors determination

The agar disc diffusion method was used to determine the resistance to the following antimicrobials (Oxoid): penicillins – amoxicillin and clavulanic acid (AMC; 20–10 µg), ampicillin (AMP; 10 µg), cephalosporins – cefoxitin (FOX; 30 µg), phenicols – chloramphenicol (C; 30 µg), fluoroquinolone – enrofloxacin (ENR; 5 µg), macrolide – erythromycin (E; 15 µg), aminoglycosides – gentamicin (CN; 10 µg) and kanamycin (K; 30 µg), carbapenems – imipenem (IPM; 10 µg) and meropenem (MEM; 10 µg), streptogramins – quinupristin/dalfopristin (QD; 15 µg), ansamycins – rifampicin (RD; 30 µg), lipoglycopeptides – teicoplanin (TEC; 30 µg), tetracyklines – tetracycline (TE; 30 µg), folate pathway antagonists – trimethoprim (W; 5 µg) and trimethoprim/sulfamethoxazole (1.25/23.75 µg). An antimicrobial resistance test was performed by inoculation of the colony suspension 0.5 McFarland on Mueller–Hinton agar plates (HiMedia Laboratories) and incubation at 35 °C for 16–18 h. The results were interpreted according to breakpoints provided by CLSI (2020) and NCCLS Document (M100-S13, M2). *S. aureus* ATCC 25,923 was used as the control strain. The MAR (multiple antibiotic resistant) index was calculated as the ratio of the number of antibiotics to which an organism is resistant to the total number of antibiotics to which the organism is exposed (Krumperman 1983).

Reptilian skin staphylococci were tested for haemolytic activity, DNAse, gelatinase, lipase, protease and amylase production, as well as for slime and biofilm production. The haemolytic test was performed on blood agar plates (Columbia agar plus 5% v/v sheep blood plates; Becton Dickinson, according to the instructions of the production company). The strains were streaked onto the plates and incubated at 37 °C for one-two days. The presence of a clear (transparent) zone around the colonies was interpreted as β-haemolysis; partial haemolysis, shown by the greenish discoloration surrounding the bacterial colony, was interpreted as α-haemolysis, and the absence of activity on red blood cells was recorded as γ-haemolysis (Pereira et al. 2009). To detect the production of DNAse enzyme, isolates were streaked on DNAse agar plates (Oxoid) according to the instructions of the production company and incubated at 37 °C for 48 h (flood plates with 1 N HCl). A clear zone around the colonies after incubation was indicative of a positive result. Gelatinase activity was detected using a medium with 12% w/v of gelatine (15 g/L Tryptone, 10 g/L yeast extract, 120 g/L gelatine from bovine skin, HiMedia). The tubes inoculated with overnight culture were incubated at 30 °C for seven days, and after 1 week, at 4 °C for at least 1 h. If the bacteria did not produce gelatinase, the medium remained solid, while the presence of sufficient gelatinase turned the medium liquid even when placed in the refrigerator (Pereira et al. 2009). Lipase test agar (10 g/L of Tryptone, 10 mL/L of Tween 80 or 20, 0.111 g/L of CaCl2, 15 g/L of agar) was used for the detection of lipase activity after one-two days incubation at 37 °C. Positive isolates produced a zone of salt precipitation around the colonies (Kumar et al. 2012). For proteases production, isolates were cultured on casein agar (50 g/L of skim milk from HiMedia, 10 g/L of agar), incubated at 37 °C for two days and checked for the presence of clear zone (casein hydrolysis) around the colony (Bertelloni et al. 2021). The Congo red agar (CRA) test was employed for the detection of slime production. Isolates were inoculated onto CRA plates (0.8 g/L of Congo red, saccharose 36 g/L, BHI, 15 g/L of agar) and incubated for 24 h at 37 °C. Slime-producing strains formed black colonies, while non-producing strains produced red colonies (Arciola et al. 2002).

### Biofilm formation assay

Biofilm production was tested using a Spectrophotometric Crystal Violet Assay according to the previously published method by Toledo-Arana et al. (2001), with slight modifications as described Bujňáková and Kmeť (2012). The biofilm formation capacity was evaluated by measuring the optical density (OD) value at λ = 570 nm in a Synergy HT Multi-Mode Microplate Reader (BioTek, Winooski, VT, USA). An average OD value and a cut-off value (ODc) (defined as three standard deviations (SD) above the mean OD of the negative control: ODc = average OD of negative control+ (3 × SD of negative control) was used for classification. The final OD value of a tested strain is expressed as the average OD value of the strain reduced by the ODc value (OD = average OD of a strain − ODc). For interpretation of the results, the strains were divided into the categories described by Stepanović et al. (2007): OD ≤ ODc = no biofilm producer; ODc < OD ≤ 2×ODc = weak biofilm producer; 2×ODc < OD ≤ 4×ODc = moderate biofilm producer; 4×ODc < OD = strong biofilm producer.

### Detection of resistance genes

After identification of the isolates, DNA was extracted from the overnight cultures using a commercial kit (QIAamp DNA Mini Kit, Qiagen, DEU). The quantity and quality of extracted DNA was analysed using a NanoDrop 2000c Spectrophotometer (Thermo Fisher Scientific, Wilmington, USA) and stored at -18 °C. The isolates which showed phenotypic antibiotic resistance were examined for the presence of antibiotic-resistance genes using polymerase chain reactions (PCRs), namely for: methicillin and beta-lactamase resistance (*mecA*, *mecC* and *blaZ*), rifampicin resistance (*rpoB*), trimethoprim resistance (*drfA* (S1), *dfrK*), tetracycline resistance (*tetB*, *tetK*, *tetM*) and erythromycin resistance (*ermC*, *msrA*). The reaction mixture for PCR in each tube contained of 1–2 µL DNA; 0.2 µL each of the 0.1–1 µM primers; 0.2 µL 1U Taq DNA polymerase; 1.5–2 µL 1.5 mM MgCl_2_; 2.5 µL 200 µM dNTP; 2.5 µL 10x reaction buffer (HOT FIREPol® DNA Polymerase, Solis BioDyne, Estonia) in a total volume of 25µL. The PCR protocol consisted of an initial denaturation step at 95 °C for 4 min, followed by 33 cycles of DNA denaturation at 95 °C for 50s, primer annealing at 50–62 °C (according to the primers) for 50 s and primer extension at 72 °C for 1 min. The primers, length of their amplified products, annealing and references are listed in Table 2. The following lab strains were used as the controls: *mecA*, *mecC, blaZ* − 290516-6; *rpoB* − 180718-7; *dfrA*, *dfrK* − 231107-8j; *tetB*, *tetK*, *tetM* − 231107-11; *ermC*, *msrA* − 231107-49.

**Table 2:** The list of primers used for detection of resistance genes in staphylococci

Gene	Primer sequences (5'–3')	Annealing (°C)	Size (bp)	Reference
*mec*A	AAAATCGATGGTAAAGGTTGGC AGTTCTGCAGTACCGGATTTGC	55	532	Strommenger et al. 2003
*mec*C	GCTCCTAATGCTAATGCA TAAGCAATAATGACTACC	50	304	Cuny et al. 2011
*bla*Z	ACTTCAACACCTGCTGCTTTC TGACCACTTTTATCAGCAACC	55	173	Martineau et al. 2000
*rpo*B	GTC GTTTAC GTT CTG TAG GTG TCA ACTTTA CGATAT GGT GTT TC	62	432	Mick et al. 2010
*dfr*A (S1)	AAG CCATTG CCA AAT AGA CG ATT CGA CTT CCC AGT TTT CG	54	247	Schwarz, personal communication
*dfr*K	GCTGCGATGGATAAGAACAG GGACGATTTCACAACCATTAAAGC	50	214	Kadlec a Schwarz 2010
*tet*B	TTG GTT AGG GGC AAG TTT TG GTA ATG GGC CAA TAA CAC CG	55	228	Ng et al. 2001
*tet*K	TCG ATA GGA ACA GCA GTA CAGCAGATCCTACTCCTT		169	
*tet*M	GTGGACAAAGGTACAACGAG CGGTAAAGTTCGTCACACAC		406	
*erm*C	CTTGTTGATCACGATAATTTCC ATCTTTTAGCAAACCCGTATTC	55	190	Martineau et al. 2000
*msr*A	TCCAATCATTGCACAAAATC AATTCCCTCTATTTGGTGGT	55	163	Martineau et al. 2000

## Results

The taxonomic analysis using MALDI-TOF spectrometry revealed 25 different bacterial species present on the skin of captive bred reptiles (Table 1). Among staphylococcal species, *S. xylosus* (*n* = 22), *S. sciuri* (*n* = 16), *S. klosii* (*n* = 6), *S. cohnii* (*n* = 3), *S. arlettae* (*n* = 2), *S. haemolyticus* (*n* = 1) and *S. warneri* (*n* = 1) were identified. Aside from staphylococci, the most frequently detected genus was *Bacillus* sp. (*B. cereus*, *B. megaterium*) and *Enterococcus* sp. (*E. faecalis*). However, Gram-negative species or genera, such as *E. coli*, *Salmonella* sp. and *Acinetobacter* sp., were also not rare. The richest bacterial community was detected in *Elaphe schrenckii*, with five different bacterial genera. In two reptile species (*Uromastyx ornata* and *Uromastyx acantihinura*) it was not possible to isolate staphylococci.

The antimicrobial resistance of isolated staphylococci (*n* = 51) was tested against 15 different antimicrobials (Table 3). All strains (51/51) were resistant to ampicillin and, with one exception, to cefoxitin (50/51). A high level of resistance was also observed for meropenem (38/51), amoxicillin-clavulanic acid (21/51) and teicoplanin (21/51). In contrast, all staphylococci were susceptible to gentamicin, kanamycin and imipenem (51/51). Multi-drug resistance (resistance to three or more classes of antimicrobial agents, Magiorakos et al. 2012) was a common finding, observed in 84.3% staphylococcal strains (43/51). The MAR index ranged from 0.20 to 0.47, with an average of 0.29 ± 0.09. The strains with phenotypic resistance to beta-lactam antibiotics were examined for the presence of the gene encoding plasmid- synthesis of penicillinases (beta-lactamases, *blaZ*) and genes encoding the chromosome type of methicillin resistance (*mecA*, *mecC*). The most frequently detected gene was *blaZ* (30/51), followed by *mecA* (2/51; *S. cohnii* and *S. kloosii*), and only one strain (*S. sciuri*) harboured the *mecC* gene. Among the trimethoprim resistant isolates, the *dfrK* gene was found in all strains (12/12) and in five strains *dfrA*(S1) was also detected. The combination of *dfrK* and *dfrA*(S1) genes was present in three strains of *S. sciuri*, one strain of *S. arlettae* and one strain of *S. kloosii*. Despite resistance to tetracycline, as determined by the detection of three genes (*tetB*, *tetK*, *tetM*), only the *tetK* gene was detected in all resistant strains (7/7). Among erythromycin resistance (*ermC*, *msrA*), we found isolates only with the *msrA* gene (4/7). The gene responsible for rifampicin resistance (*rpoB*) was detected in two out of three resistant strains.

**Table 3:** Resistance of staphylococci to antimicrobials interpreted according CLSI and NCCLS document M100-S13

Antimicrobial agent	Disk content	Sensitive	Intermediate	Resistant
Ampicillin	10 µg	-	-	51/100.0
Amoxicillin-clavulanic acid	10/20 µg	30/58.8	-	21/41.1
Cefoxitin	30 µg	1/2.0	-	50/98.0
Imipenem	10 µg	51/100.0	-	-
Meropenem	10 µg	2/3.9	11/21.6	38/74.5
Teicoplanin	30 µg	5/9.8	25/49.0	21/41.2
Gentamicin	10 µg	47/92.2	4/7.8	-
Kanamycin	30 µg	41/80.4	10/19.6	-
Tetracycline	30 µg	40/78.5	4/7.8	7/13.7
Trimetoprim	5 µg	13/25.5	26/51.0	12/23.5
Trimetroprim/sulfamethoxazole	1.25/23.75 µg	42/82.3	8/15.7	1/2.0
Chloramphenicol	30 µg	48/94.1	-	3/5.9
Rifampicin	30 µg	48/94.1	-	3/5.9
Quinupristin-dalfopristin	15 µg	13/25.5	27/52.9	11/21.6
Erytromycin	15 µg	7/13.7	37/72.6	7/13.7

Table 4 summarises the results obtained by the phenotypic characterisation. The detection of haemolytic activity on sheep blood revealed that only three positive strains (*S. haemolyticus*, *S. warneri* and *S. xylosus*) showed alpha-haemolysis. The results of microtitre plate biofilm assay showed that staphylococci from reptiles formed biofilms commonly. Biofilm formation was detected in 39 isolates (OD > 0.05, Table 4). Among them, 23/51 isolates were strongly adherent, 7/51 were moderately adherent and 9/51 were only weakly adherent. Most of the strains showing strong biofilm formation were from the species *S. xylosus* (17/21). The slime production testing on Congo red agar was observed in 44 isolates, including in all strains of *S. xylosus* (22/22) and *S. sciuri* (16/16). Regarding the investigated enzymatic activities, 14/51 produced protease, 11/51 produced lipases, 21/51 produced gelatinase and 13/51 produced DNAse. Common production of gelatinase, DNAse and protease was detected in *S. sciuri* strains (Table 4). Lipase activity was a more rarely observed property and was detected mostly in *S. xylosus* strains (10/21).

**Table 4:** Results of phenotypic tests detected in the examined staphylococci

Species	N°	Haemolysis		Protease	Lipase		Gelat	DNAse	Slime	Biofilm		
		α	γ		Tw20	Tw80				+++	++	+
*S. arlettae*	2	-	2	-	-	-	-	-	2	-	-	1
*S. cohnii*	3	-	3	-	-	-	1	-	3	-	-	3
*S. haemolyticus*	1	1	-	-	-	-	-	-	-	1	-	-
*S. kloosii*	6	-	6	-	-	-	-	-	-	1	2	1
*S. sciuri*	16	-	16	14	1	-	16	11	16	3	4	3
*S. warneri*	1	1	-	-	-	1	-	-	1	1	-	-
*S. xylosus*	22	1	21	-	10	6	4	2	22	17	1	1
Total	51	3	48	14	11	7	21	13	44	23	7	9

## Discussion

The characterisation of skin microorganisms is important for understanding how a host evolves in association with its microbial symbionts, in diagnosing illness, modelling immune system development and exploring the origins of potential zoonoses that affect humans (Ross et al. 2019). Since the skin is continually exposed to the environment, it is necessary to mention that despite samples being collected from one operation (zoo), the animals were divided in terrariums according to species. The using of litter of the same origin and the same feed according reptile species does not exclude contamination with similar microbiota from the environment to a certain extent. Nowadays, most studies deal with mammalian microbiota, and only a few skin microbiome studies have been conducted on non-mammalian vertebrates, such as avians, reptiles, amphibians and fishes. In general, a common feature of skin microbial communities seems to be low diversity at the phylum level but high diversity at the species level. Most skin bacteria fall into four different phyla: Actinobacteria, Firmicutes, Bacteroidetes and Proteobacteria (Grice and Segre 2011). In reptiles, according to our results, most of the isolated species belonged to the phylum Firmicutes (*Staphylococcus* sp., *Bacillus* sp., *Enterococcus* sp.) and Proteobacteria (*Escherichia* sp., *Pseudomonas* sp., *Salmonella* sp., *Acinetobacter* sp., *Citrobacter* sp. and *Serratia* sp.). Weitzman et al. (2018) analysed cutaneous microbiota on lizards and found Pseudomonadales, Actinomycetales, Burkholderiales, Sphingomonadales, Rhizobiales and Enterobacteriales to be the most abundant orders. The study of Cristina et al. (2022) determined *E. faecalis* as the most frequently isolated species; however, their study included 25 sick reptiles with no response to treatment. The identification of the *Salmonella* genus in *Elaphe schrenckii* confirms the fact that reptiles are well-known reservoir of *Salmonella*, species as previous studies also demonstrated (Zajac et al. 2021; Dégi et al. 2023). These poikilothermic vertebrates can be easily colonised with vertical and horizontal transfer and shed pathogens intermittently (Schroter et al. 2006). There are many reports of reptile-associated salmonellosis in humans, mostly affecting children (Bertrand et al. 2008). Contact with turtles or snakes is considered to present a particularly high risk of this infection, and an estimated 6% of human sporadic salmonellosis cases have been attributed to direct or indirect contact with reptiles, although the exact number is not known (Pees et al. 2023). Administration of antibiotics to eliminate these bacteria in reptiles has been unsuccessful and may result in the emergence of *Salmonella* bacteria that are resistant to antibiotics, which could complicate the treatment of infected persons. Therefore, the attempt to raise “*Salmonella*-free” reptiles has been unsuccessful.

Within the phylum Firmicutes, coagulase-negative staphylococci represent the major colonisers known to compete with coagulase-positive staphylococci for the same ecological niche (Gonzales et al. 2017). Our study, comprising skin samples (*n* = 40) from 17 different reptiles, showed the presence of only coagulase-negative staphylococci, with *S. xylosus* and *S. sciuri* as the most prevalent species. *S. xylosus* is a commensal of the skin and mucous membranes of humans and animals and a ubiquitous bacterium also naturally present in food. This species is virtually defined as a non-pathogenic staphylococcus, but a few strains of *S. xylosus* are related to animal and human opportunistic infections (Siqueira and Lima 2002; Won et al. 2002). *Staphylococcus sciuri* (presently renamed *Mammaliicoccus sciuri*) belongs to the genus whose name refers to the ecological niche from which these bacteria are typically isolated, being a wide variety of farm and wild mammals, as well as products derived thereof. Although *M. sciuri* is typical commensal bacteria, it has been occasionally identified as an opportunistic pathogen associated with mastitis, dermatitis and exudative epidermitis (Lu et al. 2017). Other staphylococcal species detected in the present study, such as *S. cohnii*, *S. arlettae*, *S. haemolyticus* and *S. warneri*, could also be commonly isolated from the skin of animals but with some variation in species from site to site. The example on which certain body sites displayed distinct staphylococcal community composition characterised by a higher proportion of selected species, thus indicating a site preference in humans, is *S. auricularis* which preferentially colonises the external auditory canal (Joglekar et al. 2023). In our previous study in dogs, *S. warneri* was found predominantly on back of dogs, whereas *S. haemolyticus* was on the nasal skin and chin of dogs (Štempelová et al. 2022). It seems that each animal species is colonised by its own staphylococcal composition. Indeed, a survey of 38 mammalian species determined that host order and species were the most significant influences on skin microbial communities (Ross et al. 2018). While *S. epidermidis* and *S. hominis* are typical coagulase-negative staphylococci on human skin (Otto 2010), *S. pseudintermedius* was the most frequently isolated species from canine skin (Štempelová et al. 2022).

Regarding susceptibility to antimicrobial agents, frequent resistance (> 10% of isolates) was observed for nine antimicrobials out of the fifteen tested. Multi-drug resistance (detected in 84% of our staphylococcal strains) is a global concern that is having a very bad impact on health care. Consistent with the results of other studies, high resistance was detected against penicillins (mainly to ampicillin and partially also to amoxicillin). These are antibiotics that have been widely prescribed to treat staphylococcal and streptococcal infections for many years. The resistance to penicillins is mediated by *blaZ*, the gene that encodes the predominantly extracellular enzyme β-lactamase hydrolysing the β-lactam ring. A study of Bertelloni et al. (2021) reported 82% resistance to ampicillin and 84% to amoxicillin in clinical staphylococcal isolates from different body sites, including the skin of sick dogs. Similarly, extensive analysis of a database for bacterial susceptibility to antimicrobials in birds, mammals and reptiles from the Iberian Peninsula showed very frequent multi-drug resistance (98.5% in reptiles) and resistance to penicillins (100% in most tested bacterial species, Munoz-Ibarra et al. 2022). The second most frequent resistance was observed for cefoxitin with 98% resistant strains (one sensitive *S. sciuri* strain excepted). Despite common phenotypical resistance to cefoxitin in our study, *mecA* and *mecC* were detected in only three isolates. This finding can be explained with small interpretation errors of the results (e.g. borderline MIC value of cefoxitin for different coagulase-negative staphylococci) or other resistance mechanisms, such as the presence of other mec genes (e.g. *mecD*), mutations at the genes inducing penicillin-binding protein (PBP4) overproduction (e.g. *yjbH* or *gdpP*), hyperproduction of β-lactamase, or cell wall defective forms with intrinsic β-lactam resistance (Pantosti et al. 2007). In contrast, aminoglycoside antibiotics, such as gentamicin and kanamycin, remain very effective agents against reptilian staphylococci. In our previous study, staphylococci from canine skin were also susceptible to gentamicin but more frequently resistant to tetracycline and erythromycin than the reptilian strains tested in this study (Štempelová et al. 2022). The difference in the resistance to carbapenem antibiotics (meropenem and imipenem) could be attributed to their structure and mechanism of action, which may be slightly different (Salmon-Rousseau et al. 2020). This may be why bacteria can be resistant to one carbapenem and at the same time susceptible to another carbapenem. Moreover, mechanisms of resistance differ by bacteria. The resistance of Gram-positive cocci is related to the poor affinity for some penicillin-binding proteins and the fact that they a have higher susceptibility to imipenem, while Gram-negative bacilli have a higher susceptibility to meropenem (Salmon-Rousseau et al. 2020). The presence of antibiotic-resistant bacteria in reptiles could be caused by frequent use of antibiotics in treatment. According to the antimicrobial history of reptiles included in our study, no individual has been treated except an *Iguana iguana* treated with ciprofloxacin one year ago. However, resistance genes could also be acquired from an external environment or other cohabiting animals, even from humans. Most drug-resistance genes are located on plasmids, and the spread of drug-resistance genes among microorganisms through plasmid-mediated conjugation transfer is the most common and effective way for the spread of multi-drug resistance (Tao et al. 2022). Therefore, monitoring the antimicrobial resistance of bacteria isolated from healthy skin is important. Staphylococci in particular can be a reservoir of resistance genes for pathogenic species (Rossi et al. 2020). For instance, Otto (2013) suggested that *S. epidermidis* is a reservoir of genes that, after horizontal transfer, facilitate the potential of *S. aureus* to colonise, survive during infection or resist antibiotic treatment, traits that are notably manifest in methicillin-resistant *S. aureus*. It is also necessary to mention the fact that the given dosages of antimicrobials are either extrapolated from human medicine or empirically assumed according to the reptile species, which could lead to a lack of a proper anti-infective agents for the particular pathology of reptiles. Based on the fact that antimicrobial resistance is a gradually increasing phenomenon in reptiles (Cristina et al. 2022), the completion of an antibiogram is a current and reliable method to combat resistance.

The production of different enzymes or other molecules defined as virulence effectors represents some of the weapons that bacteria have in their arsenal for host invasion. It seems the production of lipase, protease and DNAse is not a very common feature of reptilian coagulase-negative staphylococci. However, gelatinase activity was more frequent among the tested enzymes (over 40%). Proteases, lipases and gelatinases are extracellular enzymes enabling host penetration through soft tissues like epidermis and dermis. Some of the tested characteristics were associated with specific species, such as the production of gelatinase in all *S. sciuri* strains or the production of protease solely in *S. sciuri* species. When comparing to canine studies, the production of these enzymes was observed in at least half of all isolates, although the majority of strains was *S. pseudintermedius* species (Bertelloni et al. 2021; Štempelová et al. 2022). The production of DNAse was found exclusively in *S. sciuri* strains (69%) and two strains of *S. xylosus* in the present study; however, this ability is attributed especially to clinical strains of *S. aureus* (Daghistani et al. 2000). Nevertheless, Stepanović et al. (2001) tested 121 clinical and canine strains of *S. sciuri* species and showed DNAse activity to be very common among these strains (96.7% positive). The production of extracellular enzymes seems to be partially a species but especially a strain-dependent characteristic. Biofilm and slime formation assists bacteria in avoiding the host immune defence and antimicrobial therapy. Results showed that most of tested strains were found to be slime (86%) and biofilm producers (77%) in Congo red agar or microplate assay, respectively. The high ability of staphylococci to produce biofilm is connected with the character of its colonisation on skin and mucous surfaces. On the other hand, some strains are frequent causes of biofilm-associated infections and are easily able to infect any medical device that penetrates those surfaces, e.g. during surgery (Vuong and Otto 2002). As biofilms are resistant to host immune system and antibiotics, they contribute to the persistent and hard-to-treat character of staphylococcal diseases. According Silveira et al. (2021), the biofilm-forming capacity of *S. aureus* strains isolated from wild animals (mammals, birds and reptiles) achieved similarly 72.5%. The difference in biofilm production ability could also be observed for various species. Andrade et al. (2022) reported 51.0% biofilm production in *S. pseuditermedius*, 94.6% in *S. aureus* and 88.9% in *S. coagulans*, strains isolated from skin infections of companion animals.

In conclusion, present study provides an overview of the bacterial culturable species spectrum in reptiles kept under terrarium conditions, in general. Taxonomic analysis of skin microbiota from captive-bred reptiles showed the common occurrence of coagulase-negative staphylococci represented especially by the *S. xylosus* and *S. sciuri* species. Despite some of the virulence characteristics (production of enzymes) not being detected frequently, multi-drug resistance and the ability to produce biofilms seems to be a common feature of reptilian staphylococci. Therefore, caution in the application of antibiotics in these animals is necessary. In addition, the identification of *Salmonella* on the skin of some reptiles confirmed the fact that reptiles are often a natural reservoir of *Salmonella* without symptoms and can be a risk factor for illness in people. Since the elimination of these bacteria with antibiotics is ineffective, special hygiene rules should be followed when handling these animals and cleaning terrariums. The present study included only samples from a zoo; therefore, it will be useful to widen the collection of samples to include breeding households and pet shops to obtain a more comprehensive view of the situation. It will also be interesting to look at the properties of Gram-negative bacteria in detail.

## Data Availability

All data generated or analysed during this study are included in this published article.
